# Carbonic anhydrase inhibitory activity of phthalimide-capped benzene sulphonamide derivatives

**DOI:** 10.1080/14756366.2023.2235089

**Published:** 2023-07-13

**Authors:** Deepak Shilkar, Mohd Usman Mohd Siddique, Silvia Bua, Sabina Yasmin, Mrunali Patil, Ajay Kumar Timiri, Claudiu T. Supuran, Venkatesan Jayaprakash

**Affiliations:** a Department of Pharmaceutical Sciences and Technology, Birla Institute of Technology, Ranchi, India; b Department of Pharmaceutical Chemistry, Shri Vile Parle Kelavani Mandal’s Institute of Pharmacy, Dhule, India; c Neurofarba Department, Section of Pharmaceutical and Nutraceutical Sciences, University of Florence, Firenze, Italy; d Department of Pharmaceutical Chemistry, College of Pharmacy, King Khalid University, Abha, Saudi Arabia

**Keywords:** Carbonic anhydrase, phthalyl sulphamoyl derivatives, molecular docking, molecular dynamics

## Abstract

A series of phthalimide-capped benzene sulphonamides (**1**–**22**) reported by our group for dengue protease inhibitory activity have been evaluated for their carbonic anhydrase (hCA, EC 4.2.1.1) inhibitory activity against hCA I, hCA II. Compounds **1**, **3**, **10**, and **15** showed hCA I inhibition, whereas **1**, **4**, and **10** showed hCA II inhibition at nanomolar concentrations. Among these compounds, **1** displayed potent inhibitory activity against the hCA I (Ki = 28.5 nM) and hCA II (Ki = 2.2 nM), being 10 and 6 times more potent than acetazolamide, a standard inhibitor (Ki = 250 nM and 12 nM), respectively. Furthermore, this compound displayed 14-fold selectivity towards the hCA II isoform compared to hCA I. Molecular docking and MD simulations were performed to understand the atomic level interactions responsible for the selectivity of compound **1** towards hCA II.

## GRAPHICAL ABSTRACT

**Figure:**
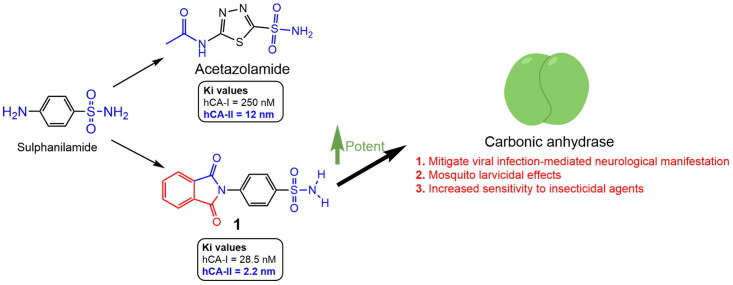

## Introduction

Carbonic anhydrases (CAs) are metalloenzymes that contain Zn^2+^ and are involved in regulating CO_2_ levels and many other physiologic processes in living organisms^1^. Eight distinct evolutionarily unrelated gene families of CAs exist: α-CAs, β-CAs, γ-CAs, δ-CAs, η-CAs, θ-CAs and ι-CAs. Among the eight subtypes, only the α-CAs are present in human cells, with 15 different isoforms identified up until now^2^^,^^3^. CAs catalyse the reversible hydration of CO_2_ to bicarbonate and protons. Several CA isoenzymes are involved in critical physiological processes, such as biosynthetic reactions^4^, acid–base regulation, gluconeogenesis, bone resorption/calcification, electrolyte secretion, and tumourigenicity^5^. Therefore, they have been well-established therapeutic targets for years. Any deregulation or dysfunction in CA activity can cause various disorders such as obesity and cancer^3^^,^^4^. Currently, several CA isoforms are essential targets for treating various disorders, including CA II, IV, XII, and XIV, which are targets for diuretics; CA II, IV, and XII for treating glaucoma and CA IX and XII isoforms as anticancer targets^5^^,^^6^.

All CA families catalyse the reversible hydration of carbon dioxide. The active site of α-CAs consists of a zinc ion coordinated to three histidine residues and a water molecule. The water molecule acts as a nucleophile and attacks the carbon dioxide molecule, forming bicarbonate and a proton. The proton is then released into the solvent through a network of hydrogen bonds involving other amino acid residues such as glutamate and asparagine. The active site of carbonic anhydrase is highly conserved among different isoforms and species, indicating its functional importance^7^. Human carbonic anhydrase II (hCA II), one of the most extensively investigated isoforms, possesses an active site comprising a zinc ion (Zn^2+^) coordinated to three histidine residues (His94, His96, and His119) and a water molecule or hydroxide ion^8^. In coordination with these residues, the zinc ion coordinated water/hydroxide serves as a nucleophilic catalyst for enzymatic activity. Apart from zinc-binding histidines, residues in proximity to the active site are involved in substrate/inhibitor orientation. Glu106 establishes a hydrogen-bond network with Thr199 that is crucial for catalysis^9–11^. Additionally, a hydrophobic pocket encompassing Val121, Leu198, and Val143 influences substrate binding and modulates enzyme function^10^.

Various studies have been performed on CA inhibitors belonging to a variety of classes. A literature survey revealed that a diverse structural class of compounds had been reported as potent CA inhibitors^1^^,^^5^^,^^6^, including sulphonamides with aromatic characteristics, specifically acting as strong CAs inhibitors^11^. Primary sulphonamides and their isosteres represent a significant class of CA inhibitors^12^, with few reports on secondary & tertiary sulphonamides^13^^,^^14^. Most of the CA inhibitors were found to have a Zinc Binding Group (ZBG) in their structures which displayed the required interactions with the Zn ion present at the active site of CAs^15^. Later many non-ZBG CA inhibitors have also been reported^16^.

Our group (Timiri et al., 2015) reported novel sulphonamides as dengue protease inhibitors^17^. Of the 20 compounds, one was found to be active with an IC_50_ value of 48.2 µM. Further optimisation was not performed because the scaffold was not a potent antiviral. Later, our team was tempted to explore this series of compounds for their ability to inhibit CA because of: (i) the presence of a sulphonamide group, an essential pharmacophore for CA inhibitors; (ii) reports and reviews on neurological manifestations associated with dengue viral infection^18^; (iii) reports on the increased level of expression of CA in the brains of patients infected with COVID-19, Dengue, and Zika viruses^19^^,^^20^; (iv) mosquito larvicidal potential of CA inhibitors^21^^,^^22^; and (iv) CAs (from insect vectors) may confer resistance to insecticides available in the market for vector control^23^. Both hCA I and hCA II and CA of *Aedes aegypti* belong to α-CAs, and hCAs inhibitors available in the market have shown some effectiveness against *A. aegypti* CA, opening a potential for their use in vector control^24^. CAs are crucial for maintaining pH homeostasis, ion transport, and other physiological processes in insects, including *A. aegypti*. Inhibiting carbonic anhydrase activity in *A. aegypti* may negatively affect the insect’s development, reproduction, or survival, thereby reducing the transmission of mosquito-borne diseases. Considering the potent inhibition of human CAs (hCA I and hCA II), it is reasonable to investigate their potential as inhibitors of *A. aegypti* carbonic anhydrase. A potential inhibitor of CA from this series will provide a starting point for optimising a candidate effective against dengue viral infection and addressing associated neurological issues. It is also expected that the effective concentration of a drug candidate in the blood of treated infected populations may also increase the sensitivity of mosquitoes (taking a blood meal from the treated infected population) to insecticides and also exert larvicidal activity.

Using a stopped-flow Applied Photophysics assay, we evaluated our previously synthesised inhibitors targeting human carbonic anhydrase isoenzymes I and II for their inhibitory activities. This assay provided valuable data on the potency and selectivity of the inhibitors, which helped assess their potential as therapeutic agents. Additionally, molecular docking and molecular dynamics simulations were employed to investigate the molecular basis of inhibition by examining the binding modes and complex stability between the inhibitors and target enzymes. These in silico analyses, combined with experimental bioassays, provide a comprehensive understanding of the inhibitory mechanisms and potential of the synthesised inhibitors as carbonic anhydrase inhibitors. These findings may facilitate the development of novel therapeutic agents targeting carbonic anhydrase and lay the groundwork for their potential cross-application in vector control.

## Material and methods

### Biological assays

The CA isoenzyme assay was performed using a stopped-flow Applied Photophysics (SX.18V-R; Oxford, UK) instrument using Khalifah’s technique^25^. hCA isoenzymes I and II used in these experiments were obtained, as earlier reported by our groups^26^. The indicator used was phenol red (0.2 mM), and the absorbance maxima at 557 nm were used in the spectrophotometric assay, working in conditions described in earlier work^27–31^.

### Molecular docking studies

The molecular docking study was conducted using AutoDock 4.2 software supplemented by MGLTools 1.5.7^32^ and AMDock utility^33^ for structure preparation and docking. Prior to docking, ligand structures were drawn and minimised using ChemOffice Professional 22.0 (PerkinElmer Informatics, Waltham, Massachusetts, USA). The proteins used in this study were human carbonic anhydrase I (PDB ID:5E2M), human carbonic anhydrase II (PDB ID:5MJN), and *A. aegypti* carbonic anhydrase II (AlphaFold, UniProt ID: AF-A0A1S4EX12). The AlphaFold structure did not contain a zinc ion present in the active site of the crystal structures obtained from the PDB. To address this, we used AlphaFill^34^ and PDB-REDO^35^ to place the zinc residue in the appropriate position, followed by a short 1 ns molecular dynamics simulation to relax the structure before docking.

The docking parameters were set as follows. A ga_pop_size of 150 was used to ensure accurate results while maintaining a reasonable computation time. A maximum of 2,500,000 energy evaluations were performed to explore sufficient conformational space for obtaining reliable results. The maximum number of generations allowed for the genetic algorithms during the docking simulation was set to 27,000 to ensure convergence and generation of reliable results. A total of 100 runs were performed, which provided a sufficient sampling of conformational space to obtain reliable results. The ligand was initially set to the bound state using the ‘unbound_model bound’ parameter, which is a common starting point for docking simulation. The docked structures were visualised using UCSF Chimaera 1.6^36^, LigPlot + v.2.2^37^, and PLIP^38^.

### Molecular dynamics simulation

For molecular dynamics simulations, the GROMACS package (version 2023.2, single precision)^39^ was used with the CHARMM36 force field^40^. The protein structure was prepared by removing water molecules and adding hydrogen atoms, followed by energy minimisation using the steepest descent algorithm. The system was solvated in a cubic box of TIP3P water molecules, with a minimum distance of 10 Å between the protein and box edges. The system was neutralised by the addition of counterions (Na^+^ or Cl^−^) using the GROMACS Genion module. The simulation was performed using an NPT ensemble with periodic boundary conditions, temperature of 300 K, and pressure of 1 atm. The equations of motion were integrated using the leapfrog algorithm, with a time step of 2 fs. Long-range electrostatic interactions were calculated using the particle mesh Ewald method, with a cut-off distance of 12 Å. The simulations were run for 300 ns, and the coordinates and velocities were saved every 10 ps for analysis.

## Results and discussion

### Chemistry

The compounds (**1**–**22**) were synthesised according to a previously reported procedure^17^.

### Biological activity

The CA inhibitory activities of all synthesised compounds 1–22 against hCA I and hCA II^41–43^ were determined using acetazolamide (ACTZ) as a reference standard (Table 1). Compounds **1**, **3, 10,** and **15** inhibited hCA I. Compound **1** was the most potent against hCA I, with a Ki value of 28.5 nM. Similarly, **1, 4,** and **10** inhibited hCA II, with **1** being the most potent against hCA II, with an enzyme Ki value of 2.2 nM. Compound **1** was a potent inhibitor of hCA I and hCA II, with 14-fold selectivity towards the hCA II enzyme and was more potent than the reference compound ACTZ (hCA I: Ki = 250 nM and hCA II: Ki = 12 nM). The study revealed ZBG-dependent inhibition of CA I and CA II enzymes because of the free sulphamoyl group in the most potent molecule, **1**. Other derivatives in which the sulphamoyl group is substituted have been reported to be inactive or poorly active.

**Table 1.** Inhibition data of CA I and CA II with compounds reported here and the standard sulphonamide inhibitor acetazolamide (**ACTZ**) by a stopped-flow CO_2_ hydrase assay.

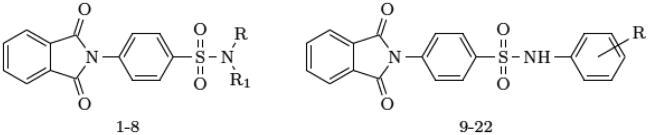				
Code	R	R_1_	Ki (nM)^a^	
			hCA I	hCA II
1	–H	–H	28.5	2.2
2	–CH(CH_3_)_2_	–H	>10,000	>10,000
3	–CH_2_–CH_2_–C_6_H_5_	–H	7352.3	>9641.0
4	–CH_3_	–CH_3_	>10,000	9062.9
5	–C_2_H_5_	–C_2_H_5_	>10,000	>10,000
6	–C_6_H_5_	–C_6_H_5_	>10,000	>10,000
7	–(CH_2_)_5_–		>10,000	>10,000
8	–(CH_2_)_2_–N(-CH_3_)–(CH_2_)_2_–		>10,000	>10,000
9	–H	–	>10,000	>10,000
10	2–OH	–	5515.8	8873.4
11	3–OCH_3_	–	>10,000	>10,000
12	4–OCH_3_	–	>10,000	>10,000
13	2–Cl	–	>10,000	>10,000
14	3–Cl	–	>10,000	>10,000
15	4–Cl	–	7497.8	>10,000
16	2–CH_3_	–	>10,000	>10,000
17	3–CH_3_	–	>10,000	>10,000
18	4–CH_3_	–	>10,000	>10,000
19	4–C_2_H_5_	–	>10,000	>10,000
20	3–NO_2_	–	>10,000	>10,000
21	4–NO_2_	–	>10,000	>10,000
22	4–COOH	–	>10,000	>10,000
ACTZ	–	–	250	12

^a^Mean from three different assays using a stopped-flow technique (errors were in the range of ± 5–10% of the reported values).

The potent CA Inhibitor, **1,** identified in this study was previously reported by our group to be inactive against dengue viral protease. The potent dengue protease inhibitor **19** reported by our group was inactive against hCA I and hCA II in the current study. Furthermore, the lead optimisation strategy should consider exploring the chemical space around the phenyl ring of phthalimide and benzene sulphonamide, keeping the sulphamoyl amino group unsubstituted for multi-target directed ligand (MTDL) design.

### Molecular docking studies

In the *in vitro* studies, compound **1** was the most potent inhibitor of hCA I and hCA II among the 22 compounds evaluated. In terms of selectivity, **1** displayed ∼10-fold more selectivity towards hCA II than towards hCA I. Accordingly, molecular docking was performed using AutoDock 4.2 to investigate the binding interactions of a small molecule ligand and reference molecule acetazolamide with its target proteins. We also conducted molecular docking studies of compound **1** against CA from *A. aegypti* to explore the potential of the compound as an inhibitor of mosquito-borne diseases such as dengue fever, Zika virus, or chikungunya. These studies could help establish a relative correlation between humans and *A. aegypti* CAs, providing insight into potential applications in vector control, which could also help develop novel strategies for combating mosquito-borne diseases.

**Figure 1.**
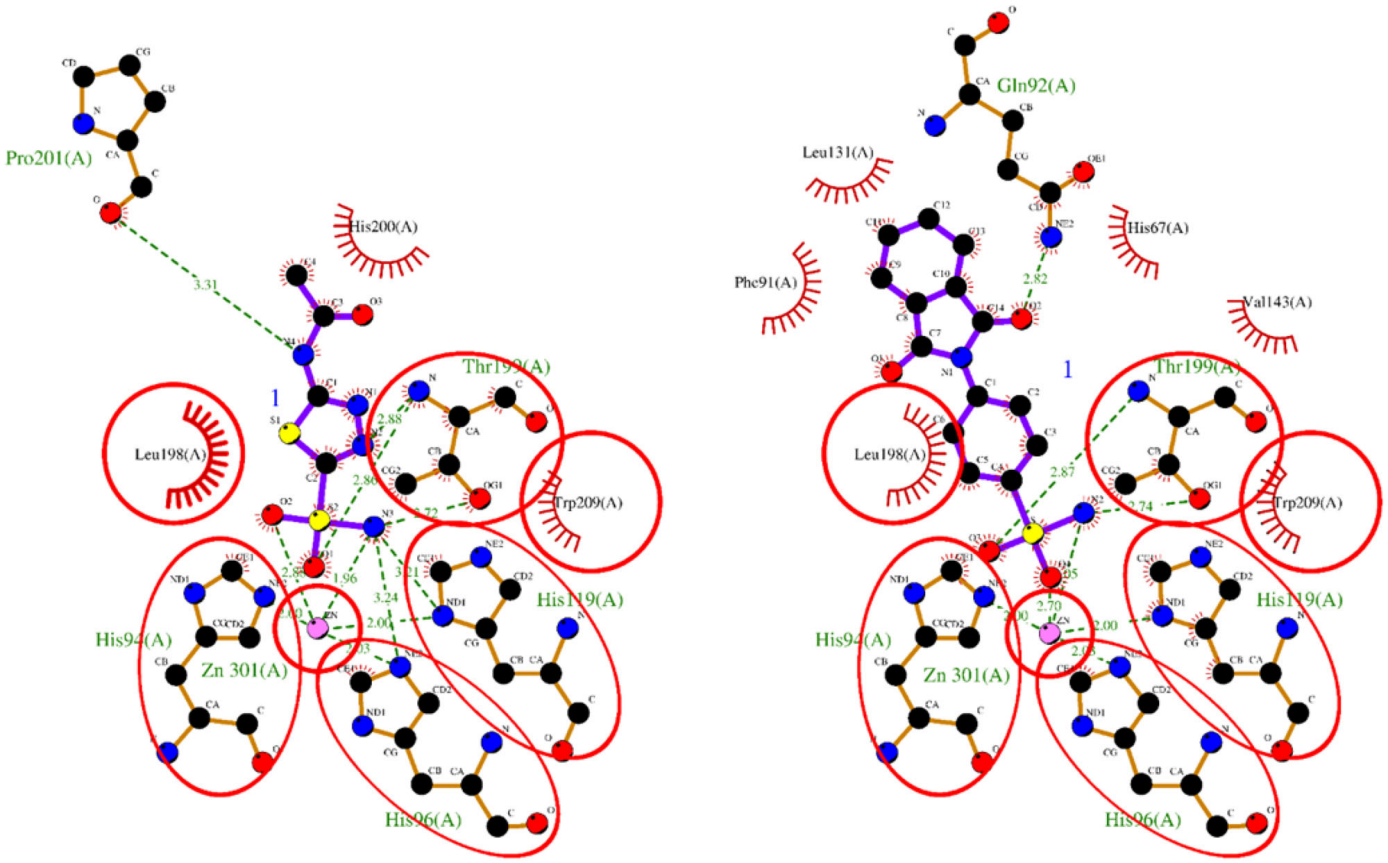
A 2D plot illustrating the interaction of ACTZ and compound **1** with human carbonic anhydrase II (PDB: 5E2M). The plot depicts the common interacting residues between the two ligands as red circles, hydrophobic interactions as arcs with red spikes, and H-bonding interactions as dashed green lines. The plot uses colour coding to distinguish between different atoms and bonds, with carbon atoms in black, oxygen atoms in red, nitrogen atoms in blue, sulphur atoms in yellow, and chloro atoms in green. The bonds of the amino acid residues are coloured brown, while the ligands are coloured violet. The zinc ion is presented in pink.

**Figure 2.**
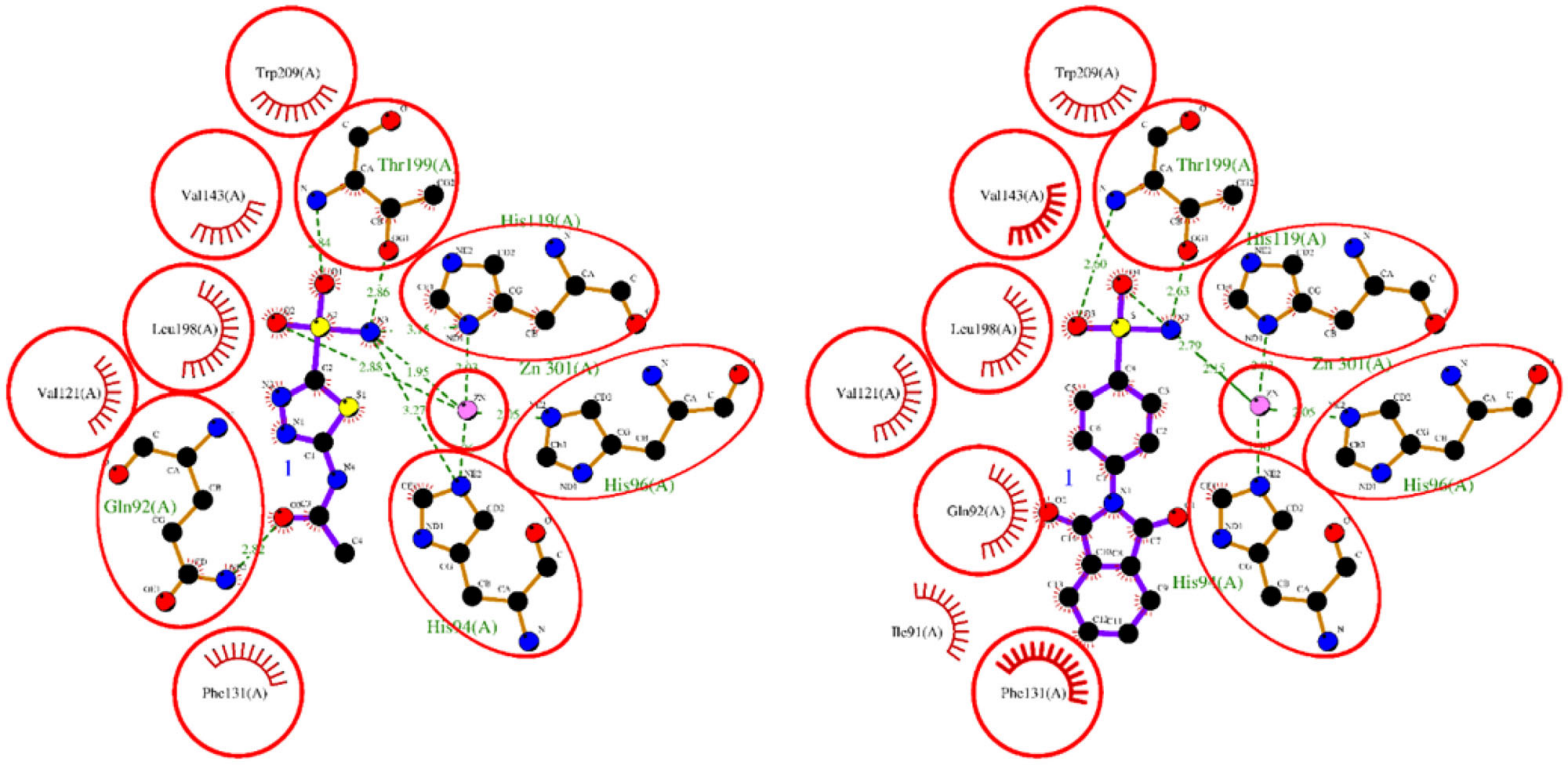
A 2D plot illustrating the interaction of ACTZ and compound **1** with human carbonic anhydrase II (PDB: 5MJN). The plot depicts the common interacting residues between the two ligands as red circles, hydrophobic interactions as arcs with red spikes, and H-bonding interactions as dashed green lines. The plot uses colour coding to distinguish between different atoms and bonds, with carbon atoms in black, oxygen atoms in red, nitrogen atoms in blue, sulphur atoms in yellow, and chloro atoms in green. The bonds of the amino acid residues are coloured brown, while the ligands are coloured violet. The zinc ion is presented in pink.

**Figure 3.**
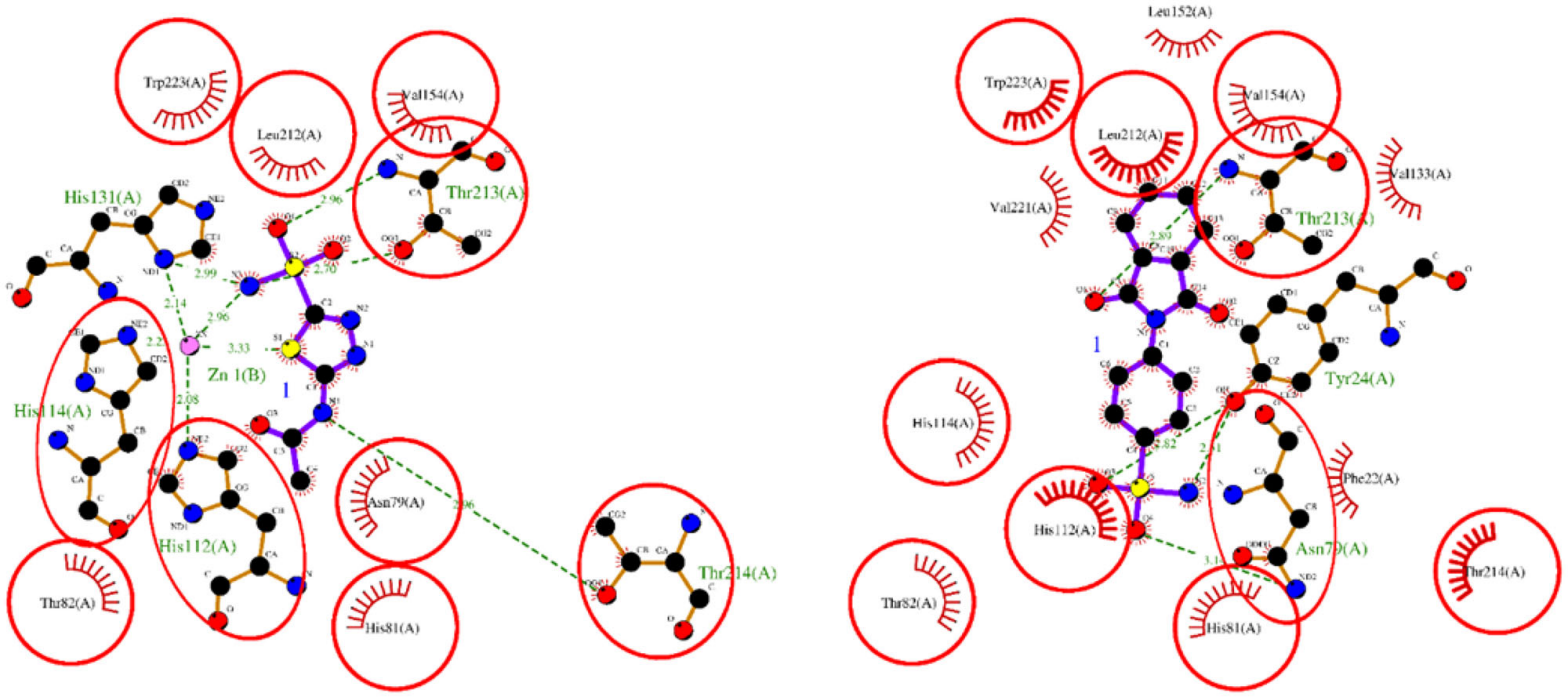
A 2D plot illustrating the interaction of ACTZ and compound **1** with AF-A0A1S4EX12 (AlphaFold structure). The plot depicts the common interacting residues between the two ligands as red circles, hydrophobic interactions as arcs with red spikes, and H-bonding interactions as dashed green lines. The plot uses colour coding to distinguish between different atoms and bonds, with carbon atoms in black, oxygen atoms in red, nitrogen atoms in blue, sulphur atoms in yellow, and chloro atoms in green. The bonds of the amino acid residues are coloured brown, while the ligands are coloured violet. The zinc ion is presented in pink.

The results of the molecular docking studies are summarised in Table 2. For hCA I, both ACTZ and **1** showed good docking scores, with the **1** having a lower score and, therefore, higher predicted affinity. Compound **1** interacted with the active site histidine residues (His94, His96, and His119), as well as other key residues such as Thr199, Gln92, Phe91, and His200. Hydrophobic interactions were observed with Leu198, Trp209, Val143, Ala121, and Phe131 (Figure 1). For hCA II, both ACTZ and **1** had similar docking scores, but **1** had a significantly lower predicted Ki value, indicating higher affinity. Compound **1** interacted with the same active site residues as hCA I, including His94, His96, His119, Thr199, and Gln92. Additionally, hydrophobic interactions were observed with Phe131, Val121, Leu198, Val143, Trp209, and Ile91 (Figure 2). For AF-A0A1S4EX12, ACTZ and **1** showed lower docking scores and predicted Ki values, indicating a lower affinity towards the protein. However, ACTZ was observed to interact with His131, Thr213, His114, Thr214, and His112, while **1** interacted with Thr213, Tyr24, Asn79, and His112. Hydrophobic interactions were observed with Trp233, Leu212, Val154, Asn79, His81, Thr82, His114, His112, Thr214, Val221, Leu152, and Trp223 (Figure 3).

**Table 2.** Comparative docking data and key residue interactions of ligands ACTZ and compound **1** with human and mosquito carbonic anhydrases.

	Molecule	Docking score (kcal/mol)	Predicted Ki	Interaction types		
				Hydrogen bonding	π–π stacking	Hydrophobic
hCA I	ACTZ	−9.43	121.99 nM	Pro201, His94, His96, His119, Thr199		Leu198, Trp209, His200
	**1**	−10.74	13.34 nM	His94, His96, His119, Thr199, Gln92	Phe91, His94	Phe91, Ala121, Leu131, Leu198, Trp209, Val143, His67,
hCA II	ACTZ	−9.37	135.03 nM	Thr199, His119, His96, His94, Gln92		Trp209, Val143, Leu198, Val121, Phe131,
	**1**	−11.13	6.9 nM	Thr199, His119, His96, His94, Gln92	Phe131	Phe131, Ile91, Gln92, Val121, Leu198, Val143, Trp209
AF-A0A1S4EX12	ACTZ	−5.74	62.25 µM	His131, Thr213, His114, Thr214, His112	His112	Trp233, Leu212, Val154, Asn79, His81, Thr82,
	**1**	−7.30	4.47 µM	Thr213, Tyr24, Asn79,	His112	His114, His112, Thr82, His81, Thr214, Val154, Leu152, Trp223, Leu212, Val221

### Molecular dynamics simulation

The biophysical interactions between docked complexes of **1** and ACTZ with hCA I and hCA II were analysed using 300 ns molecular dynamics (MD) simulation studies to validate the ligand-protein complexes. The Root Mean Square Deviation (RMSD), Root Mean Square Fluctuation (RMSF), and % interaction between the ligand and protein atoms were analysed based on the results. Additionally, the interactions observed during docking studies were verified to determine their retention over the 300 ns MD simulation. Conformational changes throughout the simulation were measured as RMSD values, with the initial frame serving as the standard backbone, and the deviation at the end was compared to the starting point. The system was stabilised and equilibrated when RMSD values ranged from 1 to 3 Å. More significant fluctuations were associated with lower complex stability.

**Figure 4.**
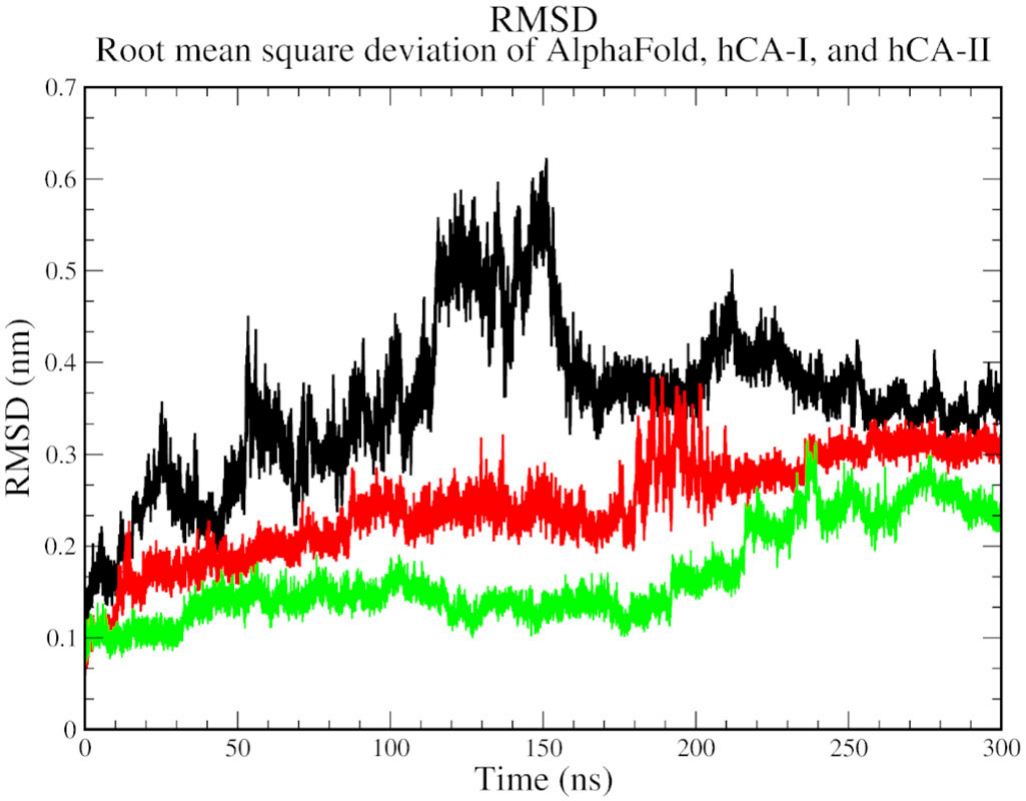
The root mean square deviation plot of AlphaFold (black), hCA I (red), and hCA II (green) with compound **1**.

The RMSD analysis revealed that all three complexes start with a high RMSD, indicating the flexibility and structural adjustments of the complexes, and gradually stabilise over time. At 100 ns, hCA I and hCA II stabilise, while the AlphaFold version of carbonic anhydrase shows a jump in RMSD. All complexes eventually stabilise at 250 ns and show a decline thereafter. The trend suggests the stability of the protein-ligand complexes over the simulation time (Figure 4).

**Figure 5.**
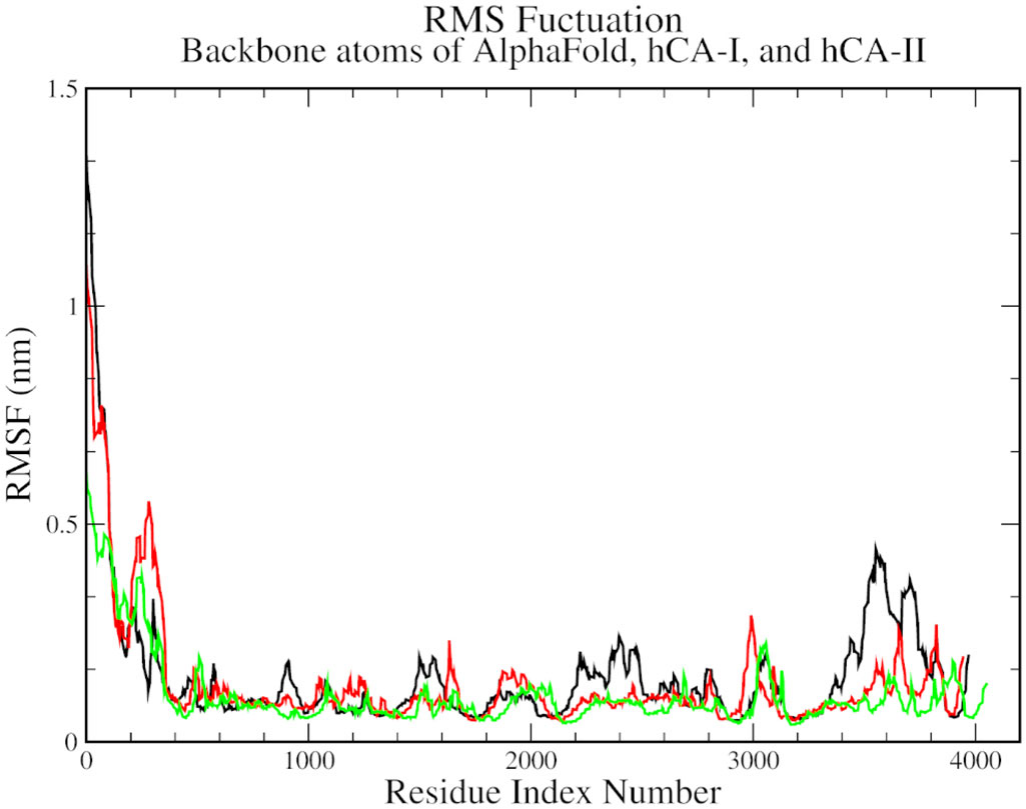
The root mean square fluctuation plot of AlphaFold (black), hCA I (red), and hCA II (green) with compound **1**.

On the RMSF plots, a significant variation was observed between C-alpha residues 110 to 130 and between 190 to 210, which was common for all three complexes. This variation could be representative of the spatial adjustment of the active site residues, especially the histidines and hydrophobic threonine and leucine residues (Figure 5).

**Figure 6.**
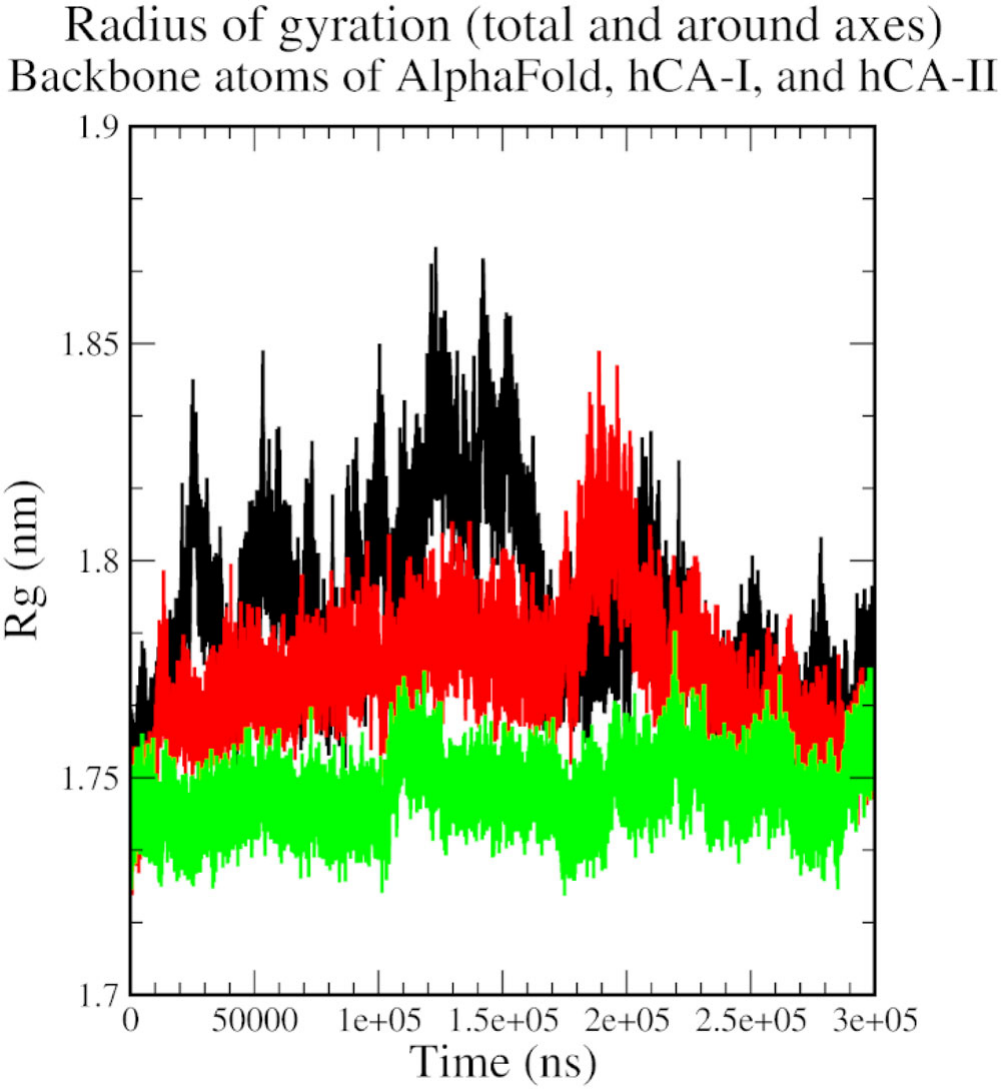
The radius of gyration plot of AlphaFold (black), hCA I (red), and hCA II (green) with compound **1**.

The radius of the gyration plot shows an initial increase in the size of the protein-ligand complex, indicating conformational changes during the simulation, followed by a reduction and stabilisation near 250 ns after a few periodical spikes, indicating some level of structural stabilisation and the formation of stable interactions between the protein and the ligand (Figure 6).

**Figure 7.**
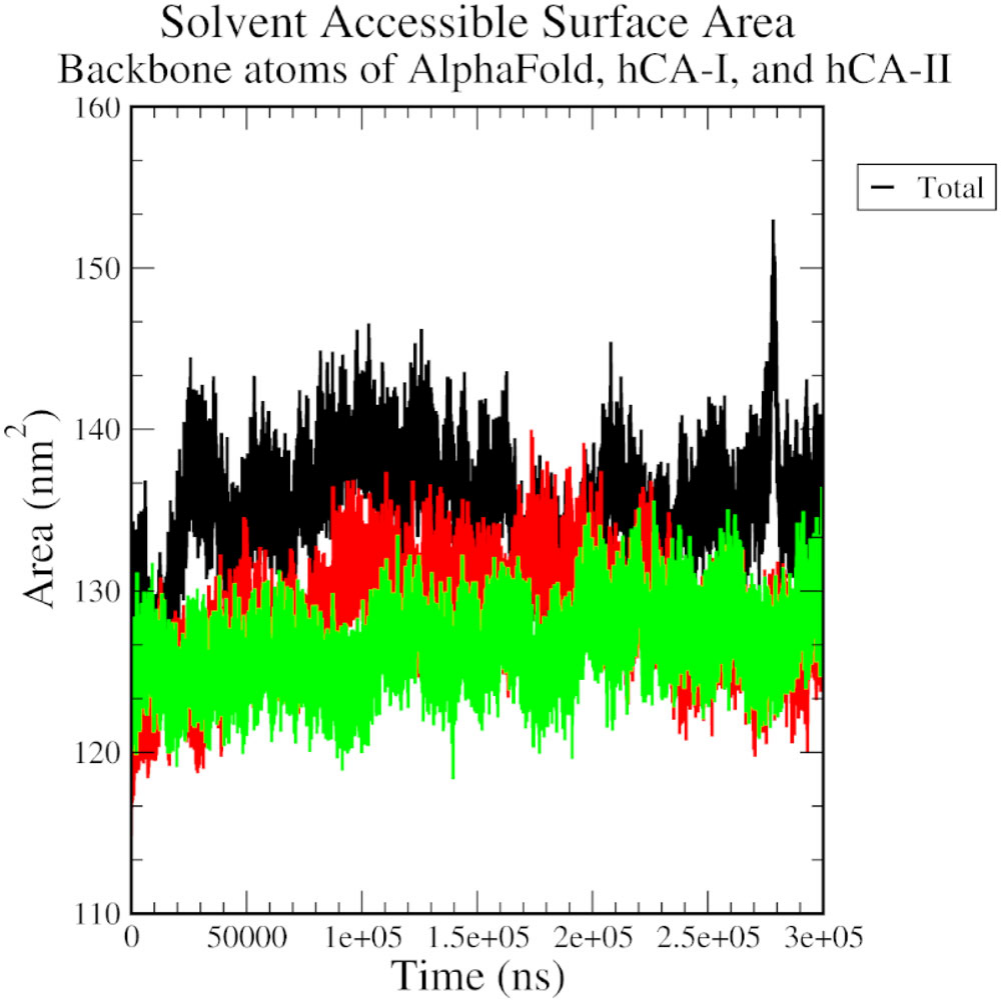
The solvent-accessible surface area plot of AlphaFold (black), hCA I (red), and hCA II (green) with compound **1**.

**Figure 8.**
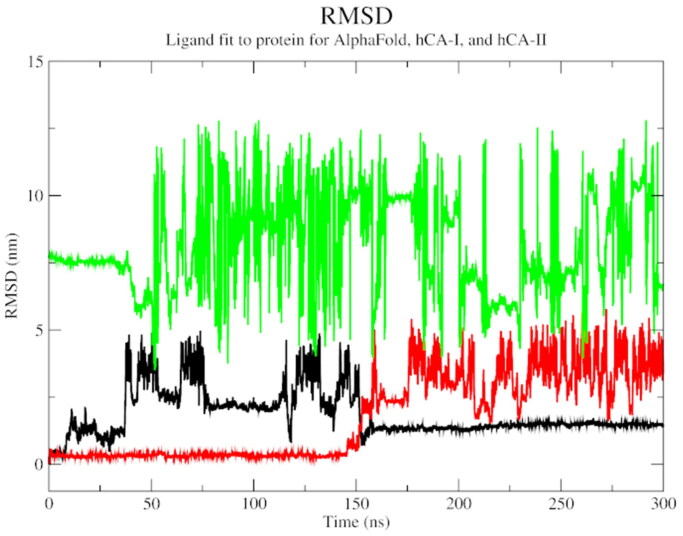
The RMSD plot of ligand fit to protein backbone of AlphaFold (black), hCA I (red), and hCA II (green) with compound **1**.

An overall increasing SASA trend for all three complexes indicated that the protein-ligand interaction becomes more solvent-exposed overtime during the simulation (Figure 7). An RMSD plot of ligand fit to protein structure revealed a significant initial variation, followed by a stable general trend, especially for the AlphaFold complex. This observation attests to the stability of the protein-ligand complex over the simulation time (Figure 8).

In this study, MD simulations of carbonic anhydrase-ligand complexes revealed fluctuations in protein-ligand interactions over time. The RMSF plots showed significant variation in the active site residues, especially the histidines and hydrophobic threonine and leucine residues, indicating their dynamic behaviour in the protein-ligand complex. These results suggest that the protein-ligand interaction in carbonic anhydrase is dynamic and fluctuates during the MD simulation. The observed correlation between the docking results, MD simulation data, predicted Ki values, and docking scores with the *in vitro* experimental data highlights the validity of the computational approach employed in this study and provides deeper insights into the molecular mechanisms. This indicates that the MD simulations effectively captured the dynamic behaviour of the protein-ligand complexes and their interactions, providing insights into the underlying molecular mechanisms. Furthermore, the strong correlation with experimental data reinforces the reliability of the predicted binding affinities and key residues involved in protein-ligand interactions.

Comparing the docking scores and predicted Ki values between hCA I and hCA II with AF-A0A1S4EX12, it is evident that both ligands exhibit higher affinities for hCA I and hCA II than for AF-A0A1S4EX12. The differences in binding affinities can be attributed to variations in the interacting residues and their types, as observed in hydrogen bonding, π–π stacking, and hydrophobic interactions. While hCA I and hCA II share a common set of histidine and threonine residues involved in hydrogen bonding, mosquito carbonic anhydrase exhibits a different pattern of interactions involving unique residues such as His131 and Thr213. Furthermore, π–π stacking interactions were observed for **1** in hCA II and AF-A0A1S4EX12 but not in hCA I. The similarity in π–π stacking interactions between compound **1** and both hCA II and AF-A0A1S4EX12, which are carbonic anhydrase II enzymes from different species, indicated the potential conservation of binding site features between these enzymes. This conservation indicated that the binding pocket of the carbonic anhydrase II isoform is structurally and functionally conserved across species, implying a similar role in their respective organisms.

In terms of drug development, conservation presents both challenges and opportunities. On the one hand, the similarity in binding interactions may make it challenging to design highly selective inhibitors that target only the mosquito carbonic anhydrase (AF-A0A1S4EX12) without affecting the human isoform (hCA II). Consequently, achieving selectivity in drug design might necessitate focusing on subtle differences in the binding sites or exploiting allosteric sites that may be unique to the mosquito enzyme. On the other hand, this conservation also provides an opportunity to leverage the extensive knowledge and understanding of human carbonic anhydrase II for drug development. Researchers can utilise existing data and establish strategies for the development of inhibitors against mosquito enzymes, potentially accelerating the discovery process. Additionally, this conservation could help to identify broad-spectrum inhibitors that target carbonic anhydrase II enzymes in multiple species, which might be useful in addressing a variety of vector-borne diseases.

## Conclusion

Our drug design hypothesis was validated by synthesising a small library of sulphonamides incorporating phthalyl moieties with free and N-substituted sulphamoyl groups (**1–22**) and screened against CA isoforms, such as CA I, which are cytosolic and associated with GI and CA II related to glaucoma. The synthesised derivatives displayed ZBG-dependent inhibition of the hCA I and hCA II enzymes. Compounds **1**, **3**, **10**, and **15** inhibited hCA I, whereas compounds **1**, **4**, and **10** inhibited hCA II. Among these compounds, **1** displayed potent inhibitory activity at low nanomolar concentrations against hCA I (Ki = 28.5 nM) and hCA II (Ki = 2.2 nM) enzymes. In hCA II, **1,** was six times more potent than ACTZ (Ki = 12 nM), and in the case of hCA I, it was 10 times more potent than ACTZ (Ki = 250 nM). Moreover, the identified lead compound displayed 14 folds selectivity towards the hCA II isoform compared to hCA I. The in silico results also confirmed a similar binding pattern for **1** and ACTZ, where the free sulphamoyl group acted as a zinc-binding group. The incorporation of hydrophobic rings increased the potency of the synthesised molecules. Compound **1** can be further optimised to obtain more potent molecules and can be used in further stages of the drug design process.

## References

[1] Agamennone M, Fantacuzzi M, Carradori S, Petzer A, Petzer JP, Angeli A, Supuran CT, Luisi G. Coumarin-based dual inhibitors of human carbonic anhydrases and monoamine oxidases featuring amino acyl and (pseudo)-dipeptidyl appendages: in vitro and computational studies. Molecules. 2022;27(22):7884. 36431985 10.3390/molecules27227884 PMC9692511

[2] Kumar S, Rulhania S, Jaswal S, Monga V. Recent advances in the medicinal chemistry of carbonic anhydrase inhibitors. Eur J Med Chem. 2021;209(:112923. 33121862 10.1016/j.ejmech.2020.112923

[3] Arechederra RL, Waheed A, Sly WS, Supuran CT, Minteer SD. Effect of sulfonamides as carbonic anhydrase va and vb inhibitors on mitochondrial metabolic energy conversion. Bioorg Med Chem. 2013;21(6):1544–1548. 22854196 10.1016/j.bmc.2012.06.053

[4] Gawad NMA, Amin NH, Elsaadi MT, Mohamed FMM, Angeli A, De Luca V, Capasso C, Supuran CT. Synthesis of 4-(thiazol-2-ylamino)-benzenesulfonamides with carbonic anhydrase i, ii and ix inhibitory activity and cytotoxic effects against breast cancer cell lines. Bioorg Med Chem. 2016;24(13):3043–3051. 27234893 10.1016/j.bmc.2016.05.016

[5] Angeli A, Carta F, Nocentini A, Winum JY, Zalubovskis R, Akdemir A, Onnis V, Eldehna WM, Capasso C, Simone G, et al. Carbonic anhydrase inhibitors targeting metabolism and tumor microenvironment. Metabolites. 2020;10(10):412. 33066524 10.3390/metabo10100412 PMC7602163

[6] Göcer H, Akıncıoğlu A, Göksu S, Gülçin İ. Carbonic anhydrase inhibitory properties of phenolic sulfonamides derived from dopamine related compounds. Arabian J Chem. 2017;10(3):398–402.

[7] Supuran CT. Carbonic anhydrases: Novel therapeutic applications for inhibitors and activators. Nat Rev Drug Discov. 2008;7(2):168–181. 18167490 10.1038/nrd2467

[8] Lindskog S. Structure and mechanism of carbonic anhydrase. Pharmacol Ther. 1997;74(1):1–20. 9336012 10.1016/s0163-7258(96)00198-2

[9] Liang Z, Xue Y, Behravan G, Jonsson BH, Lindskog S. Importance of the conserved active-site residues tyr7, glu106 and thr199 for the catalytic function of human carbonic anhydrase ii. Eur J Biochem. 1993;211(3):821–827. 8436138 10.1111/j.1432-1033.1993.tb17614.x

[10] Kim JK, Lee C, Lim SW, Andring JT, Adhikari A, McKenna R, Kim CU. Structural insights into the effect of active-site mutation on the catalytic mechanism of carbonic anhydrase. IUCrJ. 2020;7(Pt 6):985–994. 10.1107/S2052252520011008 PMC7642793 33209313

[11] Supuran CT. Carbon- versus sulphur-based zinc binding groups for carbonic anhydrase inhibitors? J Enzyme Inhib Med Chem. 2018;33(1):485–495. 29390912 10.1080/14756366.2018.1428572 PMC6009921

[12] Najm MAA, Mahmoud WR, Taher AT, Abbas SE, Awadallah FM, Allam HA, Vullo D, Supuran CT. Design and synthesis of some new benzoylthioureido phenyl derivatives targeting carbonic anhydrase enzymes. J Enzyme Inhib Med Chem. 2022;37(1):2702–2709. 36168122 10.1080/14756366.2022.2126463 PMC9542353

[13] D'Ascenzio M, Carradori S, De Monte C, Secci D, Ceruso M, Supuran CT. Design, synthesis and evaluation of n-substituted saccharin derivatives as selective inhibitors of tumor-associated carbonic anhydrase xii. Bioorg Med Chem. 2014;22(6):1821–1831. 24560739 10.1016/j.bmc.2014.01.056

[14] D'Ascenzio M, Carradori S, Secci D, Vullo D, Ceruso M, Akdemir A, Supuran CT. Selective inhibition of human carbonic anhydrases by novel amide derivatives of probenecid: synthesis, biological evaluation and molecular modelling studies. Bioorg Med Chem. 2014;22(15):3982–3988. 25027802 10.1016/j.bmc.2014.06.003

[15] Ibrahim HS, Allam HA, Mahmoud WR, Bonardi A, Nocentini A, Gratteri P, Ibrahim ES, Abdel-Aziz HA, Supuran CT. Dual-tail arylsulfone-based benzenesulfonamides differently match the hydrophobic and hydrophilic halves of human carbonic anhydrases active sites: selective inhibitors for the tumor-associated hca ix isoform. Eur J Med Chem. 2018;152:1–9. 29684705 10.1016/j.ejmech.2018.04.016

[16] Liguori F, Carradori S, Ronca R, Rezzola S, Filiberti S, Carta F, Turati M, Supuran CT. Benzenesulfonamides with different rigidity-conferring linkers as carbonic anhydrase inhibitors: an insight into the antiproliferative effect on glioblastoma, pancreatic, and breast cancer cells. J Enzyme Inhib Med Chem. 2022;37(1):1857–1869. 35768159 10.1080/14756366.2022.2091557 PMC9246135

[17] Timiri AK, Selvarasu S, Kesherwani M, Vijayan V, Sinha BN, Devadasan V, Jayaprakash V. Synthesis and molecular modelling studies of novel sulphonamide derivatives as dengue virus 2 protease inhibitors. Bioorg Chem. 2015;62(:74–82. 26247308 10.1016/j.bioorg.2015.07.005

[18] Puccioni-Sohler M, Rosadas C, Cabral-Castro MJ. Neurological complications in dengue infection: A review for clinical practice. Arq Neuropsiquiatr. 2013;71(9B):667–671. 24141501 10.1590/0004-282X20130147

[19] Zolfaghari Emameh R, Falak R, Bahreini E. Application of system biology to explore the association of neprilysin, angiotensin-converting enzyme 2 (ace2), and carbonic anhydrase (ca) in pathogenesis of sars-cov-2. Biol Proced Online. 2020;22(1):11. 32572334 10.1186/s12575-020-00124-6 PMC7302923

[20] Allgoewer K, Maity S, Zhao A, Lashua L, Ramgopal M, Balkaran BN, Liu L, Purushwani S, Arevalo MT, Ross TM, et al. New proteomic signatures to distinguish between zika and dengue infections. Mol Cell Proteomics. 2021;20:100052. 33582300 10.1016/j.mcpro.2021.100052 PMC8042398

[21] Suryawanshi RK, Patil CD, Borase HP, Narkhede CP, Salunke BK, Patil SV. Mosquito larvicidal and pupaecidal potential of prodigiosin from serratia marcescens and understanding its mechanism of action. Pestic Biochem Physiol. 2015;123(:49–55. 26267052 10.1016/j.pestbp.2015.01.018

[22] Francis SA, Taylor-Wells J, Gross AD, Bloomquist JR. Toxicity and physiological actions of carbonic anhydrase inhibitors to aedes aegypti and drosophila melanogaster. Insects. 2016;8(1):2. 28025488 10.3390/insects8010002 PMC5371930

[23] Ye W, Zhang C, Xu N, Sun Y, Ma L, Shen B, Zhou D, Zhu C. Carbonic anhydrase ii confers resistance to deltamethrin in culex pipiens pallens. Arch Insect Biochem Physiol. 2017;96(4):e21428. 10.1002/arch.21428 29086997

[24] Fisher SZ, Tariku I, Case NM, Tu C, Seron T, Silverman DN, Linser PJ, McKenna R. Expression, purification, kinetic, and structural characterization of an alpha-class carbonic anhydrase from aedes aegypti (aaca1). Biochim Biophys Acta. 2006;1764(8):1413–1419. 16920039 10.1016/j.bbapap.2006.06.013

[25] Khalifah RG. The carbon dioxide hydration activity of carbonic anhydrase. I. Stop-flow kinetic studies on the native human isoenzymes b and c. J Biol Chem. 1971;246(8):2561–2573. 4994926

[26] Krasavin M, Shetnev A, Baykov S, Kalinin S, Nocentini A, Sharoyko V, Poli G, Tuccinardi T, Korsakov M, Tennikova TB, et al. Pyridazinone-substituted benzenesulfonamides display potent inhibition of membrane-bound human carbonic anhydrase ix and promising antiproliferative activity against cancer cell lines. Eur J Med Chem. 2019;168:301–314. 30826507 10.1016/j.ejmech.2019.02.044

[27] Dilworth JR, Pascu SI, Waghorn PA, Vullo D, Bayly SR, Christlieb M, Sun X, Supuran CT. Synthesis of sulfonamide conjugates of cu(ii), ga(iii), in(iii), re(v) and zn(ii) complexes: carbonic anhydrase inhibition studies and cellular imaging investigations. Dalton Trans. 2015;44(11):4859–4873. 25711495 10.1039/c4dt03206c

[28] Ramya PVS, Angapelly S, Angeli A, Digwal CS, Arifuddin M, Babu BN, Supuran CT, Kamal A. Discovery of curcumin inspired sulfonamide derivatives as a new class of carbonic anhydrase isoforms i, ii, ix, and xii inhibitors. J Enzyme Inhib Med Chem. 2017;32(1):1274–1281. 28965419 10.1080/14756366.2017.1380638 PMC6010064

[29] Thacker PS, Alvala M, Arifuddin M, Angeli A, Supuran CT. Design, synthesis and biological evaluation of coumarin-3-carboxamides as selective carbonic anhydrase ix and xii inhibitors. Bioorg Chem. 2019;86(:386–392. 30763885 10.1016/j.bioorg.2019.02.004

[30] Supuran CT. Carbonic anhydrase inhibition and the management of neuropathic pain. Expert Rev Neurother. 2016;16(8):961–968. 27211329 10.1080/14737175.2016.1193009

[31] Mboge MY, Mahon BP, Lamas N, Socorro L, Carta F, Supuran CT, Frost SC, McKenna R. Structure activity study of carbonic anhydrase ix: selective inhibition with ureido-substituted benzenesulfonamides. Eur J Med Chem. 2017;132(:184–191. 28363153 10.1016/j.ejmech.2017.03.026 PMC5946058

[32] Morris GM, Huey R, Lindstrom W, Sanner MF, Belew RK, Goodsell DS, Olson AJ. Autodock4 and autodocktools4: automated docking with selective receptor flexibility. J Comput Chem. 2009;30(16):2785–2791. 19399780 10.1002/jcc.21256 PMC2760638

[33] Valdes-Tresanco MS, Valdes-Tresanco ME, Valiente PA, Moreno E. Amdock: a versatile graphical tool for assisting molecular docking with autodock vina and autodock4. Biol Direct. 2020;15(1):12. 32938494 10.1186/s13062-020-00267-2 PMC7493944

[34] Hekkelman ML, de Vries I, Joosten RP, Perrakis A. Alphafill: Enriching alphafold models with ligands and cofactors. Nat Methods. 2023;20(2):205–213. 36424442 10.1038/s41592-022-01685-y PMC9911346

[35] Joosten RP, Long F, Murshudov GN, Perrakis A. The pdb_redo server for macromolecular structure model optimization. IUCrJ. 2014;1(Pt 4):213–220. 10.1107/S2052252514009324 PMC4107921 25075342

[36] Pettersen EF, Goddard TD, Huang CC, Couch GS, Greenblatt DM, Meng EC, Ferrin TE. Ucsf chimera–a visualization system for exploratory research and analysis. J Comput Chem. 2004;25(13):1605–1612. 15264254 10.1002/jcc.20084

[37] Laskowski RA, Swindells MB. Ligplot+: Multiple ligand-protein interaction diagrams for drug discovery. J Chem Inf Model. 2011;51(10):2778–2786. 21919503 10.1021/ci200227u

[38] Salentin S, Schreiber S, Haupt VJ, Adasme MF, Schroeder M. Plip: fully automated protein-ligand interaction profiler. Nucleic Acids Res. 2015;43(W1):W443–447. 25873628 10.1093/nar/gkv315 PMC4489249

[39] Abraham MJ, Murtola T, Schulz R, Páll S, Smith JC, Hess B, Lindahl E. Gromacs: high performance molecular simulations through multi-level parallelism from laptops to supercomputers. SoftwareX. 2015;1–2:19–25.

[40] Huang J, Rauscher S, Nawrocki G, Ran T, Feig M, de Groot BL, Grubmuller H, MacKerell AD. Jr. Charmm36m: an improved force field for folded and intrinsically disordered proteins. Nat Methods. 2017;14(1):71–73. 27819658 10.1038/nmeth.4067 PMC5199616

[41] Taslimi P, Gulcin I, Ozgeris B, Goksu S, Tumer F, Alwasel SH, Supuran CT. The human carbonic anhydrase isoenzymes i and ii (hca i and ii) inhibition effects of trimethoxyindane derivatives. J Enzyme Inhib Med Chem. 2016;31(1):152–157. 25697270 10.3109/14756366.2015.1014476

[42] Bonardi A, Nocentini A, Bua S, Combs J, Lomelino C, Andring J, Lucarini L, Sgambellone S, Masini E, McKenna R, et al. Sulfonamide inhibitors of human carbonic anhydrases designed through a three-tails approach: improving ligand/isoform matching and selectivity of action. J Med Chem. 2020;63(13):7422–7444. 32519851 10.1021/acs.jmedchem.0c00733 PMC8008423

[43] Whyte MP. Carbonic anhydrase ii deficiency. Bone. 2023;169(:116684. 36709914 10.1016/j.bone.2023.116684

